# The Gut–Brain–Immune Axis in Environmental Sensitivity Illnesses: Microbiome-Centered Narrative Review of Fibromyalgia Syndrome, Myalgic Encephalomyelitis/Chronic Fatigue Syndrome, and Multiple Chemical Sensitivity

**DOI:** 10.3390/ijms26209997

**Published:** 2025-10-14

**Authors:** Kentaro Watai, Masami Taniguchi, Kenichi Azuma

**Affiliations:** 1 Department of Preventive Medicine and Behavioral Sciences, Kindai University Faculty of Medicine, 377-2 Ono-Higashi, Osaka-Sayama 589-8511, Osaka, Japan; 2 Clinical Research Center for Allergy and Rheumatology, NHO Sagamihara National Hospital, Sagamihara 252-0392, Kanagawa, Japan; 3 Center for Immunology and Allergy, Shonan Kamakura General Hospital, Kamakura 247-8533, Kanagawa, Japan

**Keywords:** central sensitization, fibromyalgia syndrome, gut–brain axis, microbiome, multiple chemical sensitivity, myalgic encephalomyelitis/chronic fatigue syndrome

## Abstract

Environmental sensitivity illnesses—including fibromyalgia syndrome (FMS), myalgic encephalomyelitis/chronic fatigue syndrome (ME/CFS), and multiple chemical sensitivity (MCS)—are chronic, disabling disorders characterized by hypersensitivity to environmental stimuli, persistent fatigue, widespread pain, and neurocognitive and autonomic dysfunction. Although their diagnostic criteria differ, increasing evidence suggests overlapping clinical features and shared biological mechanisms. A unifying hypothesis highlights the gut–brain–immune axis, where alterations in the intestinal microbiome, epithelial barrier dysfunction, and aberrant immune signaling interact with central sensitization and systemic metabolic dysregulation. Recent studies demonstrate reduced microbial diversity, depletion of anti-inflammatory taxa (e.g., *Faecalibacterium prausnitzii*, *Bifidobacterium*), and enrichment of pro-inflammatory *Clostridium* species across these conditions. These shifts likely alter production of short-chain fatty acids, amino acid metabolites, and complex lipids, with downstream effects on mitochondrial function, neuroinflammation, and host energy metabolism. Moreover, emerging clinical interventions—including probiotics, prebiotics, synbiotics, and fecal microbiota transplantation—suggest a potential role for microbiome-targeted therapies, though controlled evidence remains limited. This review synthesizes current knowledge on microbiome alterations in FMS, ME/CFS, and MCS, emphasizing their convergence on metabolic and immune pathways. By integrating microbial, immunological, and neurophysiological perspectives, we propose a microbiome-centered framework for understanding environmental sensitivity illnesses and highlight avenues for translational research and therapeutic innovation.

## 1. Introduction

Environmental sensitivity illnesses, including fibromyalgia syndrome (FMS), myalgic encephalomyelitis/chronic fatigue syndrome (ME/CFS), and multiple chemical sensitivity (MCS), are chronic, disabling conditions characterized by heightened sensitivity to environmental stimuli, persistent fatigue, pain, and a constellation of neurocognitive and autonomic symptoms. Despite differing diagnostic criteria and symptom profiles, these disorders share overlapping clinical features and are increasingly conceptualized within the framework of environmental sensitivity illnesses [1,2,3,4].

A central unifying mechanism proposed in recent years is central sensitization, a state of heightened responsiveness within the central nervous system (CNS) to peripheral sensory input [5,6]. Central sensitization involves enhanced synaptic transmission, diminished descending inhibition, and neuroimmune activation, resulting in an exaggerated response to normally non-noxious stimuli [7,8]. Originally described in the context of chronic pain disorders, this neurophysiological phenomenon has since been implicated in the pathogenesis of FMS, ME/CFS, and potentially MCS [9,10].

Neuroimaging studies in patients with FMS and ME/CFS have demonstrated alterations in brain regions involved in pain processing, sensory integration, and interoception, including the insula, visual cortical system, and frontal lobe [11,12,13,14]. In MCS, functional MRI and SPECT studies have revealed increased activation in response to innocuous stimuli, suggesting dysfunctional central amplification [15,16,17].

Central sensitization also appears to be tightly linked to peripheral immune and metabolic signals, including those derived from the gut microbiota [18,19]. The human gut harbors a dense and diverse microbial ecosystem that communicates bidirectionally with the central nervous system through the gut–brain axis [20,21]. This communication occurs via neural (e.g., vagus nerve), endocrine (e.g., cortisol), immune (e.g., cytokines), and metabolic (e.g., short-chain fatty acids) pathways [21,22].

**Figure 1:**
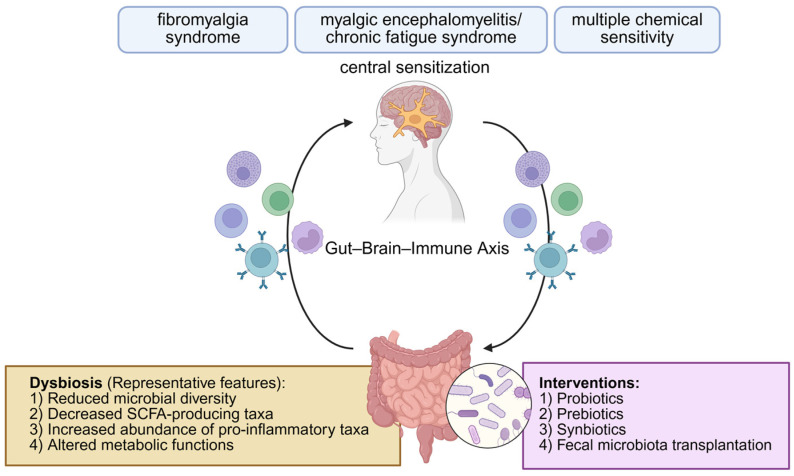
Gut–brain–immune axis in environmental sensitivity illnesses. Dysbiosis contributes to immune dysregulation and central sensitization; microbiome-targeted interventions are indicated. Created with BioRender.com.

Dysbiosis, broadly defined, refers to a disruption of the normal composition, diversity, or functional activity of the gut microbiota, leading to a loss of homeostatic balance between the host and its microbial ecosystem [23]. Such alterations may include a reduction in beneficial commensal bacteria, an overgrowth of potentially pathogenic species, or decreased microbial diversity, all of which can impair immune regulation, epithelial barrier integrity, and metabolic signaling [24]. Beyond gastrointestinal diseases such as inflammatory bowel disease, dysbiosis has been increasingly linked to systemic conditions through the gut–brain–immune axis, influencing neuroinflammation, visceral pain, fatigue, and mood disorders [25]. The human gut microbiome harbors trillions of microorganisms that collectively contribute to the production of bioactive metabolites, including short-chain fatty acids (SCFAs), bile acids, and neurotransmitter precursors. These microbial products function as critical mediators of host immunity, epithelial homeostasis, and neural activity [26]. Within this framework, dysbiosis provides a conceptual basis for understanding how gut microbial alterations may contribute to the pathophysiology of fibromyalgia syndrome, ME/CFS, and MCS. Disruption of the gut–brain axis—due to gut dysbiosis, intestinal barrier dysfunction, or altered microbial metabolites—has been implicated in the development of fatigue, mood disorders, visceral hypersensitivity, and chronic pain [27,28]. The microbiota is thus increasingly recognized not merely as a passive bystander, but as an active modulator of brain–gut–immune interactions (Figure 1).

Microbiome research commonly employs two sequencing approaches: 16S rRNA gene sequencing and shotgun metagenomic sequencing [29,30,31,32]. The 16S method amplifies variable regions of the 16S ribosomal RNA gene (e.g., V3–V4) for next-generation sequencing [30]. It is cost-effective and useful for profiling microbial community structure and diversity but generally resolves taxonomy only to the genus level, with species-level identification remaining difficult due to high sequence similarity [29,30]. Moreover, as it targets a single marker gene, functional capacity cannot be directly assessed; predictions using tools such as PICRUSt or KEGG pathway mapping remain indirect and less accurate than whole-genome-based analyses [33]. By contrast, shotgun metagenomics sequences the entire genomic content of bacteria, archaea, fungi, viruses, and eukaryotes in a sample [31,32]. This enables species- and strain-level resolution and provides direct insights into gene repertoires, including metabolic pathways (e.g., short-chain fatty acid synthesis, bile acid metabolism), antibiotic resistance genes, and virulence factors [34,35]. Although more costly and computationally demanding, shotgun metagenomics offers superior resolution and functional characterization, making it particularly valuable for mechanistic and multi-omics studies.

Probiotics are live microorganisms that confer health benefits by modulating the gut microbiota, while prebiotics are fermentable fibers that stimulate the growth of beneficial microbes, particularly short-chain fatty acids (SCFAs) producers [36]. Synbiotics are defined as mixtures of live microorganisms and substrates selectively utilized by host microorganisms that confer health benefits. They may be complementary—where probiotics and prebiotics act independently—or synergistic, in which the prebiotic specifically enhances the survival and activity of the co-administered probiotic, supporting gut microbiota balance and host health [36,37]. Nutritional interventions can be regarded as broader prebiotic approaches, as they provide complex substrates such as fibers and polyphenols that selectively promote beneficial microbial taxa. High-fiber and plant-based diets favor SCFA-producing bacteria, whereas exclusion diets such as low-FODMAP may reduce fermentable substrates and offer symptomatic relief in subsets of patients [38,39]. Thus, nutritional modulation can be viewed as an extension of prebiotic strategies within microbiome-targeted interventions.

In this review, we summarize and compare current findings on the gut microbiome in FMS, ME/CFS, and MCS across five major domains: (1) microbial diversity; (2) taxa increased; (3) taxa decreased; (4) functional alterations; and (5) intervention outcomes. By integrating insights from taxonomic and functional analyses within a gut–brain–immune framework, we aim to clarify the extent to which dysbiosis represents a shared pathophysiological signature and to inform future directions in diagnostics and therapeutics among environmental sensitivity illnesses.

This review was conducted as a narrative review rather than a registered systematic review. Therefore, the search strategy was not deposited on PROSPERO or similar registries.

## 2. Methods

### 2.1. Search Strategy

A comprehensive literature search was conducted to identify studies examining microbiome alterations in FMS, ME/CFS, and MCS. The search was performed in PubMed and Google Scholar in July 2025. PubMed served as the primary database to identify articles meeting the search criteria, while Google Scholar was used as a supplementary source. The query combined disease-specific and microbiome-related terms: “microbiome” in “fibromyalgia,” “chronic fatigue syndrome,” or “multiple chemical sensitivity.” In addition, to capture interventional evidence, a parallel search was performed using the same disease-specific terms combined with the keyword “intervention” (i.e., “intervention” in “fibromyalgia,” “chronic fatigue syndrome,” or “multiple chemical sensitivity”). This combined strategy yielded 498 records, which were exported for subsequent screening and eligibility assessment.

### 2.2. Screening and Eligibility Criteria

Titles and abstracts were screened against predefined inclusion and exclusion criteria. Studies were considered eligible if they met all of the following conditions:

1. Human subjects: Clinical populations were studied, rather than animal or in vitro models.
2. Target conditions: Participants had a confirmed diagnosis of FMS, ME/CFS, or MCS. Studies of overlapping but distinct syndromes (e.g., chronic pain not meeting fibromyalgia syndrome criteria) were excluded.
3. Sample size: Each study arm included at least five participants.
4. Microbiome analysis: Validated methodologies such as 16S rRNA gene sequencing, shotgun metagenomics, metatranscriptomics, quantitative PCR, metabolomics, or culture-based approaches were employed.
5. Outcomes: At least one microbiome-related endpoint was reported, including microbial composition, alpha or beta diversity, abundance of specific taxa, or functional/metabolic pathways.
6. Study design: Observational (cross-sectional, cohort, or case–control) or interventional studies were eligible.

Conference abstracts without microbiome data, case reports, animal studies, and reviews without primary synthesis were excluded.

Eligibility was assessed holistically, considering all criteria simultaneously to minimize the risk of omitting relevant studies.

### 2.3. Data Extraction

Data were extracted from eligible studies using a standardized framework, and outputs were independently reviewed by investigators for accuracy and completeness.

### 2.4. Extracted Data Included:

1. Study design: Type (cross-sectional, case–control, cohort, interventional) and methodological features (e.g., multi-omic approaches, machine learning).
2. Participant characteristics: Sample size, case–control distribution, diagnostic categories, demographics (age, sex distribution), and disease duration, when available.
3. Microbiome analysis methods: Sequencing platforms (e.g., Illumina, 454), targeted rRNA regions (V3/V4, V4), metagenomics or metatranscriptomics, metabolomics, and immune profiling.
4. Microbiome findings: Changes in microbial diversity (alpha, beta), abundance of specific taxa, functional or metabolic pathway alterations, and directionality of change (increased, decreased, divergent).
5. Clinical correlations: Associations between microbiome features and clinical outcomes, including symptoms (fatigue, pain, cognitive dysfunction, psychological distress, gastrointestinal symptoms) or biomarkers (e.g., inflammatory cytokines, bile acids, short-chain fatty acids).

Titles and abstracts were screened independently by two investigators (KW and MT). Full-text eligibility and data extraction were conducted in duplicate. Disagreements were resolved through discussion, and when consensus was not achieved, adjudication was provided by a third investigator (KA). All reviewers are medical scientists with training in environmental medicine and clinical research.

### 2.5. Flow of Study Selection

**Figure 2:**
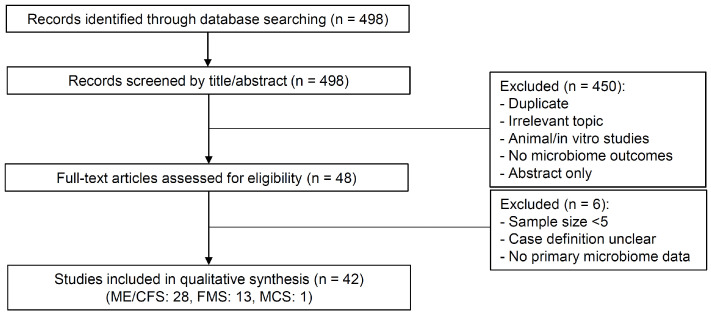
Flow diagram showing the literature search and study selection process. ME/CFS, myalgic encephalomyelitis/chronic fatigue syndrome; FMS, fibromyalgia syndrome; MCS, multiple chemical sensitivity.

The literature search identified 498 records. After duplicate removal, all records proceeded to title and abstract screening, during which 450 records were excluded due to irrelevance, non-human models, or lack of microbiome outcomes. A total of 48 full-text articles were assessed for eligibility. Of these, 6 articles were excluded because they did not meet the predefined inclusion criteria (e.g., insufficient sample size, undefined case definition, lack of primary microbiome data). Ultimately, 42 studies were included in the review: 28 on ME/CFS, 13 on FMS, and 1 on MCS (Figure 2).

## 3. Microbial Diversity in Environmental Sensitivity Illnesses

### 3.1. Microbial Diversity and Clinical Relevance

The gut microbiome represents a highly complex and dynamic ecosystem that plays an essential role in host health through immune regulation, metabolic functions, and maintenance of gut barrier integrity. Greater microbial diversity is generally considered beneficial, as it reflects ecological stability and functional redundancy, which contribute to resilience against perturbations such as infection, dietary change, or antibiotic exposure [24,40]. Reduced diversity, conversely, has been associated with multiple pathological conditions, including inflammatory bowel disease, obesity, and neuroimmune disorders [23,41].

### 3.2. Diversity Metrics: Alpha and Beta Diversity

Microbial diversity is commonly assessed using two principal dimensions: alpha diversity and beta diversity.

Alpha diversity describes within-sample diversity, encompassing both species richness (the number of taxa present) and evenness (the relative distribution of taxa). Common indices include the Shannon index, Simpson’s index, and observed species count. High alpha diversity indicates a rich and balanced microbial community, often correlated with metabolic versatility and gut ecosystem stability [42].

Beta diversity refers to between-sample diversity, capturing compositional dissimilarities among microbial communities from different individuals or conditions. Metrics such as Bray–Curtis dissimilarity or UniFrac distances are widely used to visualize these differences via ordination methods like principal coordinates analysis (PCoA). Altered beta diversity patterns often signal disease-specific microbiome signatures and can distinguish patient populations from healthy controls [43,44].

### 3.3. Alpha Diversity in Environmental Sensitivity Illnesses

Most primary studies included in recent reviews have reported a reduction in alpha diversity among individuals with FMS and ME/CFS compared to healthy controls. This finding indicates a loss of microbial richness and/or evenness, potentially reflecting a narrowed repertoire of beneficial commensals and a reduced capacity for metabolic resilience. In FMS, several studies have reported significant reductions in Shannon and Chao1 indices, indicating compromised ecological stability of the gut microbiota [45,46,47]. Similarly, patients with ME/CFS exhibit decreased alpha diversity across multiple independent cohorts [48,49,50].

The reduction in alpha diversity among FMS and ME/CFS patients may contribute to compromised production of key microbial metabolites, such as short-chain fatty acids (SCFAs), and has been linked to fatigue severity, pain perception, and markers of systemic inflammation [51,52].

### 3.4. Beta Diversity in Environmental Sensitivity Illnesses

In addition to reduced alpha diversity, significant alterations in beta diversity have been observed in FMS and ME/CFS populations, suggesting that the overall microbial community structure differs consistently between patients and controls. Studies using principal coordinate analysis (PCoA) of Bray–Curtis dissimilarity or UniFrac distances revealed clear clustering patterns, indicating disease-specific microbial signatures [47]. Notably, the compositional separation in beta diversity appears to be more pronounced in early-stage ME/CFS, highlighting potential temporal dynamics in microbial shifts [50,53].

By contrast, findings for MCS differ. A recent metagenomic study reported no significant differences in beta diversity (Bray–Curtis) between MCS patients and healthy controls, suggesting that while the overall gut community structure remains relatively similar, MCS is characterized by more subtle taxonomic shifts rather than broad compositional divergence [54].

## 4. Taxonomic Shifts in the Gut Microbiome at the Phylum, Genus, and Species Levels

The major taxa that are consistently reported as increased or decreased across FMS, ME/CFS, and MCS are summarized in Table 1, together with their functional and clinical relevance.

### 4.1. Increased Taxa

***Akkermansia muciniphila*** (↑ in MCS, FMS):

Known for degrading mucin in the gut lining, *Akkermansia* is often associated with metabolic health. However, in excess, it may disrupt the mucus barrier, particularly under inflammatory conditions, potentially leading to increased gut permeability [45,54,55].

***Clostridium sensu stricto*** (↑ in ME/CFS, FMS):

Members of this group include both commensals and pathogenic species. Some produce D-lactic acid, which has been hypothesized to contribute to cognitive symptoms in ME/CFS [48,56] and FMS [45].

***Bacteroides*** (↑ in MCS, ↓ in FMS, ME/CFS):

Increased *Bacteroides* species may contribute to altered bile acid metabolism and carbohydrate fermentation [57]. Some *Bacteroides* strains may also produce immunogenic polysaccharides.

In MCS, specific *Bacteroides* species, including *Bacteroides vulgatus*, *Bacteroides dorei*, and *Bacteroides ovatus*, were enriched in patients compared to healthy controls [54]. In contrast, patients with FMS and ME/CFS exhibited a significantly reduced abundance of *Bacteroides* species [45,58]. These taxa are involved in polysaccharide degradation and short-chain fatty acid production, but their overrepresentation may influence immune signaling and gut barrier function under dysbiotic conditions [59].

***Veillonella*** (↑ in MCS):

*Veillonella* metabolizes lactate into propionate and acetate. Its increased abundance, as reported in MCS, may reflect altered fermentation dynamics and shifts in SCFAs profiles, although its clinical significance remains uncertain [54].

**Table 1:** Altered gut microbial taxa in environmental sensitivity illnesses.

Taxa (Genus/Species)	Direction of Change	Reported in	Functional/Clinical Relevance
*Akkermansia muciniphila*	↑	FMS, MCS [45,54]	Mucin degradation; excess may compromise gut barrier
*Clostridium sensu stricto*	↑	FMS, ME/CFS [45,48]	Includes D-lactate producers; implicated in cognitive symptoms
*Bacteroides* spp. *(B. vulgatus*, *B. dorei*, *B. ovatus*, *B. uniformis)*	↑	MCS [54]	Polysaccharide degradation, bile acid metabolism; overabundance may alter immune signaling
	↓	FMS, ME/CFS [45,58]	
*Veillonella* spp.	↑	MCS [54]	Lactate fermentation to propionate/acetate; altered SCFA balance
*Faecalibacterium prausnitzii*	↓	FMS, ME/CFS, MCS [45,46,50,53,54]	Keystone butyrate producer; depletion linked to impaired epithelial integrity and systemic inflammation
*Eubacterium rectale*	↓	FMS, ME/CFS [45,50]	Major butyrate producer; loss reduces anti-inflammatory capacity
*Roseburia* spp.	↓	FMS, ME/CFS [45,48]	Butyrate synthesis; depletion associated with fatigue and pain severity
*Bifidobacterium* spp.	↓	FMS [46]	Fiber fermentation; acetate/lactate production; reduced levels may impair immune tolerance

FMS, fibromyalgia syndrome; MCS, multiple chemical sensitivity; ME/CFS, myalgic encephalomyelitis/chronic fatigue syndrome; ↑ indicates taxa enriched in patients compared with healthy controls; ↓ indicates taxa depleted in patients compared with healthy controls.

### 4.2. Decreased Taxa

***Faecalibacterium prausnitzii*** (↓ in FMS, ME/CFS, MCS):

A keystone butyrate-producing bacterium with anti-inflammatory properties, *F. prausnitzii* is consistently depleted across all three conditions [45,46,50,53,54]. Its loss is associated with impaired intestinal barrier function and lower SCFAs availability, particularly butyrate, which modulates immune and neuroendocrine function [60].

***Eubacterium rectale*** (↓ in ME/CFS, FMS):

Also a major butyrate producer, *E. rectale* plays a critical role in maintaining colonic homeostasis. Its reduction is linked to decreased anti-inflammatory SCFAs production and lower microbial metabolic resilience [45,50].

***Roseburia* spp.** (↓ in FMS, ME/CFS):

Roseburia contributes to butyrate synthesis and mucosal health. Its depletion is correlated with fatigue severity and chronic pain symptoms [45,48].

***Bifidobacterium* spp.** (↓ in FMS):

Although often used as probiotics, *Bifidobacterium* levels are reduced in FMS [46]. *Bifidobacterium* ferment dietary fibers into acetate and lactate and help suppress intestinal inflammation [61].

The recurring depletion of SCFA-producing taxa—including *Faecalibacterium*, *Eubacterium*, *Roseburia*, and *Coprococcus*—suggests a shared dysbiotic pattern in FMS and ME/CFS, characterized by impaired epithelial integrity, systemic inflammation, and altered neuromodulatory signaling. On the other hand, the enrichment of *Streptococcus*, *Veillonella*, and *Clostridium* may reflect fermentative imbalances, local acidosis, or shifts in gut pH and redox potential. While these patterns are more robustly established in ME/CFS and FMS, similar trends are emerging in MCS, although fewer studies are available.

## 5. Functional Alterations in Host–Microbiome Metabolic Interactions

In the following sections, we summarize functional alterations revealed by gut microbiome profiling and host-targeted metabolomic analyses. Importantly, these results represent an integrated view, reflecting both microbial functional capacities and host metabolic phenotypes, which together shape the pathophysiology of FMS, ME/CFS, and MCS.

### 5.1. Functional Analysis in Fibromyalgia Syndrome

Accumulating evidence from multi-omics approaches—including 16S rRNA sequencing, shotgun metagenomics, serum metabolomics, and targeted biochemical assays—indicates that FMS is associated not only with compositional shifts in the gut microbiota but also with profound functional alterations that may influence host metabolism, immune responses, and pain modulation. Key functional alterations in microbial pathways observed in FMS are summarized in Table 2.

#### 5.1.1. Short-Chain Fatty Acids Metabolism

Several studies have reported a reduction in butyrate- and propionate-producing bacteria (e.g., *Faecalibacterium prausnitzii*, *Eubacterium* spp.) in FMS patients, accompanied by decreased serum butyrate and propionate concentrations [45,47]. These SCFAs are known to exert anti-inflammatory and neuromodulatory effects, suggesting that their depletion may contribute to systemic inflammation and altered pain processing in FMS [26].

**Table 2:** Functional alterations in fibromyalgia syndrome.

Pathway/Metabolism	Key Findings	Clinical/Functional Relevance
Short-chain fatty acid metabolism [45,47]	Reduced abundance of butyrate- and propionate-producing bacteria (*Faecalibacterium prausnitzii*, *Eubacterium* spp.); decreased serum SCFAs	Loss of anti-inflammatory and neuromodulatory effects; may promote systemic inflammation and pain
Neurotransmitter metabolism [46,62]	Altered glutamate metabolism; reduced abundance of *Bifidobacterium* and *Eubacterium*; elevated circulating glutamate	Excess glutamate implicated in central sensitization and chronic pain
Bile acid metabolism [63]	Altered secondary bile acids (e.g., depletion of α-muricholic acid); changes in bile acid-metabolizing bacteria	Correlated with pain intensity and fatigue; link between bile acid dysregulation and nociplastic pain
Energy and carbohydrate metabolism [64]	Elevated metabolites related to energy/carbohydrate metabolism; altered host- and gut-derived metabolites	Suggests disturbances in microbial–host metabolic pathways influencing fatigue and pain

SCFAs, short-chain fatty acids.

#### 5.1.2. Neurotransmitter Metabolism

Metabolomic profiling has revealed altered glutamate metabolism in FMS, with reduced abundance of genera (*Bifidobacterium*, *Eubacterium*) involved in neurotransmitter biotransformation [46]. Increased circulating glutamate levels are of particular interest, as glutamate is a key excitatory neurotransmitter implicated in central sensitization and chronic pain [62].

#### 5.1.3. Bile Acid Metabolism

Targeted bile acid profiling has shown significant alterations in secondary bile acids (notably, depletion of α-muricholic acid) in FMS patients, alongside shifts in bile acid-metabolizing bacterial taxa [63]. These changes correlated strongly with symptom severity, including pain intensity and fatigue, suggesting a mechanistic link between bile acid dysregulation and nociplastic pain.

#### 5.1.4. Energy Metabolism and Gut–Brain Axis Interactions

In FMS patients, significantly elevated metabolites were mainly associated with energy and carbohydrate metabolism, as well as with metabolites derived from both host and gut. These findings suggest disturbances in microbial and metabolic pathways relevant to the gut–brain axis [64]. Alterations in carbohydrate and amino acid metabolism could reflect microbial contributions to fatigue and widespread pain.

### 5.2. Functional Analysis in Myalgic Encephalomyelitis/Chronic Fatigue Syndrome

A comprehensive overview of the disrupted metabolic pathways and their clinical implications in ME/CFS is provided in Table 3.

#### 5.2.1. Butyrate Synthesis and Shifts in Carbohydrate and Lactate Metabolism

Pathway analysis using HUMAnN3 and GOmixer identified significant depletion of genetic modules involved in bacterial butyrate synthesis, particularly within the acetyl-CoA, pyruvate, and glutarate pathways. This depletion was most prominent in genes encoding butyryl-CoA–acetate CoA-transferase, the dominant terminal enzyme in the acetyl-CoA pathway. In parallel, modules related to lactate utilization and certain polysaccharide fermentation pathways were reduced, whereas pathways for lactate production, sugar metabolism, and mucin degradation were relatively enriched in ME/CFS [50,53,58].

#### 5.2.2. Amino Acid and Energy Metabolism Pathways

Functional inference from metagenomic prediction in the oral microbiome has been explored. Here, “PICRUSt-based functional inference” refers to a predictive approach in which 16S rRNA gene profiles are computationally mapped to reference genome databases (e.g., KEGG) using the PICRUSt software, thereby estimating the potential functional gene content of the microbial community rather than directly measuring it. Using this method, altered taxa were predominantly associated with amino acid and energy metabolism pathways. These functional shifts may influence systemic physiological processes, providing a plausible mechanistic link between oral dysbiosis and CFS symptomatology [58,65].

#### 5.2.3. Lipid Biosynthesis and Vitamin B6 Pathways

ME/CFS patients exhibited a reduction in unsaturated fatty acid biosynthesis pathways and an increase in atrazine degradation pathways, both of which were independent of irritable bowel syndrome (IBS) comorbidity. Additionally, enhanced vitamin B6 biosynthesis/salvage pathways and increased pyrimidine ribonucleoside degradation pathways were observed in both the IBS-negative subgroup and the overall ME/CFS cohort. These findings suggest that ME/CFS is characterized by distinct alterations in bacterial metabolic capacity, including disruptions in lipid biosynthesis and enrichment of specific degradation and biosynthetic processes, which may have implications for host nutrient metabolism and overall disease pathophysiology [66].

#### 5.2.4. Choline, Carnitine, and Complex Lipid Metabolism

Altered plasma levels of choline, carnitine, and complex lipid metabolites in ME/CFS patients, with elevated ceramide concentrations specifically in those with IBS comorbidity. Furthermore, integration of fecal metagenomic and plasma metabolomic data yielded a more accurate predictive model for ME/CFS than either analysis alone [67].

#### 5.2.5. Sphingolipid, Phospholipid, Purine, Cholesterol, Amino Acid, and Mitochondrial Metabolism

Abnormalities in 20 metabolic pathways in ME/CFS patients, with approximately 80% of diagnostic metabolites decreased, consistent with a hypometabolic syndrome. The affected pathways included sphingolipid, phospholipid, purine, cholesterol, microbiome-related, pyrroline-5-carboxylate, riboflavin, branched-chain amino acid, peroxisomal, and mitochondrial metabolism [68].

#### 5.2.6. Peroxisomal Dysfunction and Lipid/Tricarboxylic Acid Cycle Abnormalities

ME/CFS patients had significantly decreased levels of plasmalogens, phospholipid ethers, phosphatidylcholines, and sphingomyelins, along with elevated levels of dicarboxylic acids. These findings are consistent with peroxisomal dysfunction and dysregulation of lipid remodeling and the tricarboxylic acid cycle [69].

### 5.3. Functional Analysis in Multiple Chemical Sensitivity

Metagenomic functional profiling of the gut microbiota in patients with MCS has revealed distinct alterations compared with healthy controls [54]. In particular, microbial pathways associated with the degradation of environmental pollutants, including xylene and dioxin degradation, were significantly enriched, indicating a potentially heightened microbial capacity to metabolize exogenous toxic chemicals. Similarly, pathways related to antimicrobial resistance, such as the two-component system, antimicrobial resistance genes, and cationic antimicrobial peptide resistance, were also increased in abundance, suggesting an altered microbial resilience to environmental stressors. In contrast, pathways involved in amino acid metabolism and biosynthesis were markedly depleted in MCS. These included glycine, serine, and threonine metabolism, arginine biosynthesis, and aminoacyl-tRNA biosynthesis, as well as pathways related to vitamin and cofactor metabolism. Moreover, neurotransmission-related pathways, such as those linked to GABAergic and glutamatergic synapses, were also reduced [54]. Taken together, these findings suggest that the gut microbiota of MCS patients is characterized by a dual profile: an enhanced functional potential for processing exogenous chemicals and resisting antimicrobial pressure, coupled with impaired metabolic capacity for amino acids and related host–microbe signaling functions. Such alterations may contribute to immune dysregulation, disruption of the gut–brain axis, and heightened sensitivity to environmental exposures observed in MCS. Distinct functional alterations in MCS, particularly enrichment of xenobiotic degradation pathways and depletion of amino acid metabolism, are summarized in Table 4.

## 6. Studies on Microbiome-Targeted and Nutritional Approaches in Environmental Sensitivity Illnesses

An overview of interventional approaches in FMS, ME/CFS, and MCS—including fecal microbiota transplantation, probiotics, synbiotics, and nutritional strategies—is summarized in Table 5.

### 6.1. Fecal Microbiota Transplantation

Fecal microbiota transplantation (FMT) involves transferring stool from a healthy donor into the gastrointestinal tract of a patient, with the aim of restoring a healthy microbiome ecosystem. While primarily used for recurrent *Clostridioides difficile* infection, it has been investigated in conditions involving gut–brain axis dysregulation [70].

In FMS, a study by Cai et al. transplanted fecal samples from FMS patients into germ-free mice. The recipient mice exhibited increased mechanical pain sensitivity and depression-like behaviors, suggesting a causal relationship between the altered FMS microbiota and nociplastic pain [71]. In an open-label trial involving female patients with FMS, FMT of a healthy microbiota was associated with reduced pain and improved quality of life [71].

In ME/CFS, evidence from a cohort of patients, many with comorbid IBS, demonstrated that manipulation of the colonic microbiota through infusion of non-pathogenic bacteria achieved notable clinical benefits, with approximately 70% of patients responding initially and more than half maintaining sustained improvement, including long-term remissions lasting up to two decades [72]. In a retrospective study of 42 ME/CFS patients, Kenyon et al. found that FMT produced significantly greater symptom improvement than oral therapies consisting of nutritional remedies, probiotics, prebiotics, dietary advice, and lifestyle modifications [73].

In MCS, no trials have yet evaluated FMT. MCS is frequently accompanied by medication intolerance/hypersensitivity: clinical reports document positive skin tests to multiple drug classes, and survey data indicate that 60% of patients report difficulty using medicinal drugs [74]. Accordingly, the common FMT practice of administering oral antibiotics as pre-treatment may be difficult to tolerate in this population [75].

FMT in chronic non-infectious conditions raises safety and durability concerns, and donor selection remains a critical variable [76].

### 6.2. Probiotics and Nutritional Intervention in Myalgic Encephalomyelitis/Chronic Fatigue Syndrome

A pilot clinical trial investigated the effects of a multi-strain probiotic formulation—*Lactobacillus paracasei* ssp. *paracasei* F19, *Lactobacillus acidophilus* NCFB 1748, and *Bifidobacterium lactis* Bb12—on fatigue, health status, and physical activity in ME/CFS. Fifteen participants with high baseline fatigue severity and disability scores underwent a two-week observation phase without treatment, followed by a four-week probiotic intervention and a four-week follow-up. The intervention was associated with improvements in neurocognitive function, although no statistically significant changes were observed in fatigue severity or physical activity scores [77]. In an open-label pilot trial, Wallis et al. targeted gut dysbiosis in ME/CFS patients with elevated *Streptococcus* counts using a 4-week alternating regimen of erythromycin and a D-lactic acid–free multi-strain probiotic (5 × 10^10^ CFU twice daily). The intervention produced large reductions in *Streptococcus* viable counts and improvements in several clinical outcomes, including sleep quality, attention, processing speed, cognitive flexibility, memory, and verbal fluency, although fatigue and mood remained unchanged. Microbiota shifts—such as increases in *Bacteroides* and *Bifidobacterium* and decreases in *Clostridium* (notably in males)—were associated with some of the observed clinical benefits [78]. Several probiotic formulations, including *Enterococcus faecium* with *Saccharomyces boulardii* (Enterelle^®^), various *Bifidobacterium species* (Bifiselle^®^), *Bifidobacterium longum* AR81 (Rotanelle), *Lactobacillus casei* with *Bifidobacterium lactis* (Citogenex^®^), and *Lactobacillus rhamnosus* GG with *Lactobacillus acidophilus* (Ramnoselle^®^), have been investigated in patients with ME/CFS. This study suggests that probiotic supplementation may enhance overall well-being while modulating inflammatory and oxidative stress markers, with no significant adverse effects reported [79]. In a randomized, double-blind, placebo-controlled pilot trial of 39 ME/CFS patients, daily supplementation with *Lactobacillus casei* strain Shirota for two months increased fecal *Lactobacillus* and *Bifidobacterium* levels and significantly reduced anxiety symptoms compared with placebo [80].

A randomized, double-blind, crossover pilot trial investigated the effects of high cocoa liquor/polyphenol-rich chocolate (HCL/PR) versus low polyphenol iso-calorific chocolate in 10 patients with ME/CFS. Eight weeks of HCL/PR intake significantly improved fatigue scores, residual function, and mood, whereas the control chocolate worsened these outcomes. These findings suggest that cocoa polyphenol-rich chocolate may reduce symptom burden in ME/CFS patients [81]. Systematic review evaluated dietary and nutritional interventions for ME/CFS [82]. Seventeen studies investigating 14 different interventions were included, but most showed no therapeutic benefit. Improvements in fatigue were reported for nicotinamide adenine dinucleotide (NADH), and NADH combined with coenzyme Q10 (CoQ10) [83,84]. Overall, evidence was insufficient due to small sample sizes, short study durations, and methodological limitations, highlighting the need for further research in homogeneous ME/CFS populations [85].

### 6.3. Probiotics, Prebiotics, Synbiotics, and Nutritional Intervention in Fibromyalgia Syndrome

Roman et al. conducted a double-blind, placebo-controlled, randomized pilot trial to examine the effects of a multispecies probiotic on cognitive function and emotional symptoms in patients with FMS. The study assessed pain, disease impact, quality of life, anxiety and depressive symptoms, and also included computerized cognitive tasks evaluating impulsive choice and decision-making, along with urinary cortisol measurement, before and after intervention. Their findings indicated that probiotic supplementation led to improvements in impulsivity and decision-making, suggesting partial enhancement of prefrontal executive functions. However, the effects on other cognitive domains as well as emotional and somatic symptoms were limited, and the authors emphasized the need for broader investigation [86]. In a subsequent study, Cardona et al. carried out an eight-week pilot randomized controlled trial in 31 patients diagnosed with FMS, comparing multispecies probiotics with placebo for their effects on memory and attention [87]. Results demonstrated that probiotic treatment improved attentional performance by reducing errors in an attention task, particularly showing a tendency to decrease omission errors in Go trials during the Go/No-Go task—indicative of enhanced sustained attention and response control. However, no significant effects were observed on memory function. The authors noted that this attentional improvement aligned with their previous findings on reduced impulsivity, further supporting the potential cognitive benefits of probiotics in FMS. Taken together, these two pilot studies—despite their small sample sizes—suggest that probiotics may contribute to improvements in certain cognitive functions, particularly impulsivity control and attentional processes, in patients with FMS. The findings are consistent with the theoretical framework of gut microbiota–brain axis modulation influencing neurocognitive outcomes.

A one-month intervention with the synbiotic Gasteel Plus^®^ (*Bifidobacterium lactis* CBP-001010, *Lactobacillus rhamnosus* CNCM I-4036, and *Bifidobacterium longum* ES1, as well as fructooligosaccharides) in women with FMS, with or without ME/CFS, reduced perceived stress, anxiety, and depression, while improving quality of life. The supplementation also activated the hypothalamic–pituitary–adrenal axis, potentially counteracting elevated baseline IL-8 levels. No adverse effects on body composition, sleep, or activity patterns were observed [88].

A randomized clinical trial evaluated the SYNCHRONIZE+ intervention, a brief multidisciplinary program including nutrition, chronobiology, and physical activity, in patients with FMS and ME/CFS. The intervention significantly improved Mediterranean diet adherence, nutritional quality, and dietary intake patterns, with increased consumption of fruits, vegetables, legumes, nuts, fish, and fermented foods, and reduced intake of sweets, red/processed meat, and butter. It also increased protein and iron intake, reduced night eating, and improved skeletal muscle mass index, with benefits maintained over 12 months [89]. Moreover, observational and interventional studies suggest that adherence to the Mediterranean diet is associated with improvements in pain, fatigue, mood, body composition, and quality of life [90,91]. Similarly, anti-inflammatory dietary patterns, including those enriched with extra-virgin olive oil, have been shown to alleviate clinical symptoms such as pain, fatigue, and sleep disturbances, although effects on inflammatory biomarkers remain inconsistent [92,93,94]. Furthermore, low-antigen diets, particularly those reducing histamine or incorporating elimination approaches, appear to improve gastrointestinal manifestations and certain systemic symptoms, with partial benefits on pain [95]. Collectively, these findings indicate that nutritional strategies—whether based on Mediterranean, anti-inflammatory, or low-antigen principles—may contribute to symptomatic relief and enhanced well-being in patients with FMS [96].

### 6.4. Probiotics, Prebiotics, and Nutritional Intervention in Multiple Chemical Sensitivity

No controlled trials of probiotics or prebiotics have been conducted in MCS to date. Although dietary restriction is frequently practiced in clinical management of MCS [97,98,99], formal dietary intervention studies are lacking.

**Table 5:** Interventional and observational studies on microbiome and nutritional approaches in environmental sensitivity illnesses.

Disease	Participants (*n*)	Intervention Dose/Regimen	Duration	Main Outcomes
**Fecal microbiota transplantation (FMT)**				
FMS [71]	4 mice with FMS fecal vs. 4 with healthy fecal	Germ-free mice colonized with FMS fecal microbiota (*n* = 4) vs. healthy fecal microbiota (*n* = 4)	Single FMT	The recipient mice exhibited increased mechanical pain sensitivity and depression-like behaviors
ME/CFS (±IBS) [73]	21 FMT vs. 21 oral treatment	FMT: 10 separate implants per patient (different donors); Oral treatment: Probiotics (*Lactobacillus*, *Bifidobacterium*, *S. boulardii*); Prebiotics (FOS, inulin, psyllium); Nutritional supplements (vitamins, minerals, omega-3); Mediterranean-style diet and lifestyle advice	10 implants (interval not specified)	FMT group showed greater improvement in fatigue and gastrointestinal symptoms; no serious adverse events.
**Probiotics**				
ME/CFS [77]	15 (Control: Baseline self-control)	Probiotic yogurt (*L. paracasei* F19, *L. acidophilus*, *B. lactis*) 200 mL twice daily (1 × 10^8^ CFU/mL)	4 weeks	No significant change in fatigue or physical activity; modest improvement in selected neurocognitive measures.
ME/CFS [78]	44 (Control: Baseline self-control)	Erythromycin 400 mg, twice daily (week-on); D-lactate-free probiotic 5 × 10^10^ CFU, twice daily (week-off); alternated weekly for 4 weeks.	4 weeks	Reduced *Streptococcus* abundance; improved sleep and cognition; fatigue unchanged.
ME/CFS [79]	14 (Control: Baseline self-control)	Stepwise probiotic regimen: Week 1: Enterelle^®^ (*E. faecium*, *S. boulardii*) 2 capsules twice daily; Week 2: Bifiselle^®^ (*B. longum*, *B. breve*, *B. bifidum*, *B. infantis*) 2 capsules twice daily; Week 3: Ramnoselle^®^ (*L. rhamnosus* GG, *L. acidophilus*) 2 capsules twice daily; Weeks 4–8: Enterelle^®^ 2 capsules/day + Citogenex^®^ (*L. casei*, *B. lactis*) 2 capsules/day + Rotanelle^®^ (*B. longum* AR81) 2 capsules/day.	8 weeks	Improved Quality of life (QoL), mood, oxidative stress markers; no adverse events reported.
ME/CFS [80]	22 probiotic, 17 placebo	*L. casei* Shirota 24 × 10^9^ CFU/day Control: Placebo drink (identical appearance/taste, no probiotics)	8 weeks	Reduced anxiety vs. placebo; increased *Lactobacillus*/*Bifidobacterium*.
FMS [86]	23 probiotic, 21 placebo	Multi-strain probiotic (*L. rhamnosus* GG, *L. casei*, *L. acidophilus*, *L. bulgaricus*, *B. breve*, *B. longum*, *S. thermophilus*), 4 capsules/day	8 weeks	Improved decision-making; limited effects on mood.
FMS [87]	15 probiotic, 16 placebo	ERGYPHILUS Plus® (*L. rhamnosus* GG, *L. casei*, *L. acidophilus*, *B. bifidum*), 4 capsules per day	8 weeks	Reduced attentional errors; no effect on memory.
**Synbiotics**				
FMS (±CFS) [88]	15 (Control: Baseline self-control)	Synbiotic Gasteel Plus® (*B. lactis* CBP-001010, *L. rhamnosus* CNCM I-4036, *B. longum* ES1 + fructooligosaccharides), 1 stick/day (1 × 10^9^ CFU)	30 days	Improved Fibromyalgia Impact Questionnaire-Revised (FIQR), fatigue, and trait anxiety; immuno-neuroendocrine markers improved.
**Nutritional approach**				
ME/CFS [81]	10 Crossover design	High cocoa liquor/polyphenol-rich chocolate (HCL/PR chocolate) Iso-caloric, low-polyphenol chocolate as comparator 8 weeks each with 2-week washout	8 weeks	Improved fatigue, function, and mood with HCL/PR; control chocolate worsened outcomes.
ME/CFS [83]	36 treatment, 37 placebo	coenzyme Q10 200 mg/day + nicotinamide adenine dinucleotide (NADH) 20 mg/day vs. placebo	8 weeks	Reduced fatigue, improved oxidative stress markers; safe and well tolerated.
ME/CFS [84]	14 treatment, 12 placebo	NADH 10 mg/day vs. placebo	4 weeks	Improved fatigue and function; safe and well tolerated.
FMS [91]	50 intervention, 50 control	Personalized Mediterranean diet Diet counseling Control: General balanced diet counseling	8 weeks	Improved pain, fatigue, function, and pain-related anxiety.
FMS [93]	30 (female) Crossover design	Extra virgin olive oil (EVOO), 50 mL/day Control: Refined olive oil (ROO), 50 mL/day, 3 weeks; crossover with a 2-week washout	3 weeks	EVOO reduced RBC and ESR; differences in cortisol and Platelet Distribution Width.
FMS [94]	22 intervention, 24 control	Anti-inflammatory diet (AID) plus low-FODMAP for 1 month, then AID alone for an additional 2 months (total of 3 months). Control: Habitual diet, 3 months	3 months	Improved FIQR, pain, fatigue, gastrointestinal symptoms, sleep, QoL; biomarkers unchanged.
FMS [95]	40 (Control: Baseline self-control)	Histamine-release test–guided individualized elimination diet.	6 months	Improved digestive symptoms and weight; no effect on pain or Fibromyalgia Impact Questionnaire.
FMS (±CFS) [89]	27 intervention, 27 control	SYNCHRONIZE application plus brief nutritional counseling to improve Mediterranean-diet adherence. Control: Usual care only (no app, no counseling)	12 weeks	Improved Mediterranean-diet adherence, nutritional quality, and intake patterns; clinical outcomes not assessed.
FMS [90]	181 (FMS patients, online survey)	Mediterranean diet adherence (observational, Mediterranean diet adherence assessed)	Not applicable (cross-sectional survey)	Negative correlation between Mediterranean-diet adherence and disease severity.
FMS [92]	95 (female)	None (observational; Dietary Inflammatory Index, DII scores calculated) Control: None (cross-sectional, within-group comparison by DII levels)	Not applicable (cross-sectional survey)	Higher DII scores associated with lower pressure pain thresholds.

FMS: fibromyalgia syndrome; ME/CFS: myalgic encephalomyelitis/chronic fatigue syndrome; FMT: fecal microbiota transplantation; CFU: colony-forming units; QoL: quality of life; FIQR: fibromyalgia impact questionnaire-revised; NADH: nicotinamide adenine dinucleotide; EVOO: extra virgin olive oil; ROO: refined olive oil; DII: dietary inflammatory index.

## 7. Discussion

Comparative analysis of gut microbiome studies across FMS, myalgic ME/CFS, and MCS reveals both convergent and divergent microbial features that may help explain shared clinical phenotypes as well as condition-specific manifestations.

Shared microbial signatures are evident across these environmental sensitivity illnesses. A recurring finding is the consistent depletion of SCFA-producing taxa, particularly *Faecalibacterium prausnitzii*, *Eubacterium rectale*, and *Roseburia* spp. [45,46,48,50,54]. These commensals are central to butyrate synthesis, intestinal barrier maintenance, and immune tolerance. Their loss has been linked to increased systemic inflammation, impaired epithelial integrity, and disrupted neuroimmune communication, which may contribute to fatigue, nociplastic pain, and heightened stress sensitivity. The parallel depletion of beneficial *Bifidobacterium* spp., especially in FMS [46,61], further underscores a general reduction in immunoregulatory capacity across these conditions. Taken together, these findings suggest that impaired SCFAs metabolism constitutes a unifying pathophysiological axis in environmental sensitivity illnesses.

Despite these commonalities, disease-specific microbial patterns distinguish the three conditions. In FMS, reduced alpha diversity and robust shifts in beta diversity are consistently reported, highlighting an overall contraction of microbial richness and stability [45,47]. Functional studies in FMS emphasize alterations in glutamate metabolism, bile acid profiles, and carbohydrate/energy metabolism [46,62,63,64], all of which intersect with mechanisms of central sensitization and chronic pain. ME/CFS similarly shows reduced microbial diversity, but its functional signature is broader, encompassing deficits in butyrate synthesis, disturbances in amino acid and lipid metabolism, and evidence of systemic hypometabolism [48,49,50,53,66,68,69]. In contrast, MCS exhibits subtler taxonomic deviations, with no significant beta diversity shifts, but distinct enrichment of microbial pathways for xenobiotic degradation and antimicrobial resistance [54]. These unique features likely reflect the heightened environmental reactivity characteristic of MCS, pointing to microbial adaptation to exogenous chemical exposures at the expense of host-relevant metabolic functions such as amino acid and neurotransmitter synthesis.

From a mechanistic perspective, these differences suggest that while FMS and ME/CFS are primarily characterized by metabolic insufficiency and impaired neuromodulatory signaling, MCS may involve a reorientation of the microbiome toward detoxification and resilience against environmental stressors, concomitant with a loss of capacity to sustain amino acid metabolism and gut–brain axis signaling. The consistent depletion of *Faecalibacterium prausnitzii* and related SCFA-producers across all three conditions, however, supports the concept of a shared dysbiotic core, onto which disease-specific adaptations are superimposed [60]. Clinically, these convergent and divergent findings may help explain both the overlapping symptomatology (e.g., fatigue, pain, cognitive dysfunction) and the heterogeneity observed among patients. They also provide a rationale for microbiome-targeted therapies: interventions aimed at restoring SCFA-producing taxa may address common mechanisms, while strategies tailored to modulate glutamate or bile acid metabolism (in FMS), energy and lipid metabolism (in ME/CFS), or detoxification pathways (in MCS) may be required for condition-specific benefit. Future studies should therefore prioritize longitudinal, multi-omics approaches capable of disentangling causal from compensatory microbial shifts, with the goal of refining therapeutic strategies and stratifying patients according to microbiome-derived endotypes.

This narrative review has several limitations. First, it is not a systematic review or meta-analysis, and therefore the synthesis of evidence is qualitative and may be subject to selection bias. Second, most available studies were conducted in limited geographic regions, particularly Europe, North America, and Japan, which constrains the generalizability of the findings; evidence for multiple chemical sensitivity (MCS) remains especially scarce. Third, methodological heterogeneity across studies—including differences in sequencing platforms (16S rRNA versus shotgun metagenomics), sample sizes, and diagnostic criteria—limits the comparability of results. In addition, the majority of studies are cross-sectional, precluding causal inferences between dysbiosis and clinical phenotypes. Fourth, while associations between microbiome alterations and symptom profiles are increasingly recognized, the direct clinical relevance remains uncertain, and interventional trials are few, small in scale, and of short duration; no controlled intervention studies have been conducted for MCS. Finally, publication bias cannot be excluded, as positive findings are more likely to be reported. Taken together, these limitations underscore the need for larger, longitudinal, multi-omics studies and rigorously designed clinical trials to establish causal links and therapeutic potential.

## 8. Conclusions

Environmental sensitivity illnesses such as MCS, FMS, and ME/CFS present with complex, multisystemic symptoms that remain difficult to treat. Increasing evidence implicates the gut microbiota as a key modulator in their pathophysiology, particularly through its interactions with the immune, metabolic, and nervous systems via the gut–brain–immune axis. Microbial profiling may aid in stratifying patient subtypes, predicting treatment response, and identifying novel therapeutic targets. Future research should prioritize longitudinal, multi-omics studies and well-controlled interventional trials to clarify causal relationships and optimize microbiota-based therapies in this underserved patient population.

## Figures and Tables

**Table 3:** Functional alterations in myalgic encephalomyelitis/chronic fatigue syndrome.

Pathway/Metabolism	Key Findings	Clinical/Functional Relevance
Butyrate synthesis, Carbohydrate and lactate metabolism [50,53,58]	Depletion of bacterial butyrate synthesis modules (acetyl-CoA, pyruvate, glutarate pathways); reduced butyryl-CoA–acetate CoA-transferase genes	Reduced butyrate capacity; linked to fatigue and impaired immune regulation
	Reduced modules for lactate utilization and polysaccharide fermentation; enrichment of lactate production and mucin degradation	Altered fermentation and lactate accumulation may contribute to fatigue and gut symptoms
Amino acid and energy metabolism [58,65]	PICRUSt-based inference: altered amino acid and energy pathways	Suggests systemic effects of oral/gut dysbiosis on host metabolism
Lipid biosynthesis and vitamin B6 pathways [66]	Reduced unsaturated fatty acid biosynthesis; enhanced vitamin B6 biosynthesis/salvage and pyrimidine degradation	May affect host nutrient metabolism and disease pathophysiology
Choline, carnitine, and complex lipid metabolism [67]	Altered plasma choline, carnitine, ceramide; integrated microbiome–metabolome models improved diagnostic prediction	Links gut microbiome changes to systemic lipid metabolism and clinical phenotypes
Mitochondrial and peroxisomal metabolism [68]	Abnormalities in >20 pathways: reduced sphingolipid, phospholipid, purine, cholesterol metabolism; decreased plasmalogens and phosphatidylcholines; increased dicarboxylic acids	Consistent with hypometabolic syndrome and peroxisomal dysfunction

**Table 4:** Functional alterations in multiple chemical sensitivity.

Pathway/Process	Key Findings	Clinical/Functional Relevance
Degradation of environmental pollutants [54]	Xylene and dioxin degradation pathways enriched	Indicates heightened microbial potential to metabolize exogenous toxic chemicals
Antimicrobial resistance [54]	Two-component system, antimicrobial resistance genes, cationic antimicrobial peptide resistance increased	Suggests increased microbial resilience to environmental stressors
Amino acid metabolism and biosynthesis [54]	Glycine, serine, threonine metabolism; arginine biosynthesis; aminoacyl-tRNA biosynthesis decreased	Impaired microbial capacity for amino acid metabolism, potentially affecting host–microbe interactions
Vitamin and cofactor metabolism [54]	Pathways related to vitamin and cofactor metabolism reduced	Suggests limited microbial contribution to essential cofactor availability
Neurotransmission-related pathways [54]	GABAergic and glutamatergic synapse-related pathways decreased	May contribute to impaired gut–brain axis signaling and heightened sensitivity

GABA, gamma-aminobutyric acid.

## Data Availability

Not applicable.

## Abbreviations

CFU	colony-forming units
CoQ10	coenzyme Q10
DII	dietary inflammatory index
EVOO	extra virgin olive oil
FIQR	fibromyalgia impact questionnaire-revised
FMS	fibromyalgia syndrome
FMT	fecal microbiota transplantation
HPA	hypothalamic–pituitary–adrenal axis
IBS	irritable bowel syndrome
MCS	multiple chemical sensitivity
ME/CFS	myalgic encephalomyelitis/chronic fatigue syndrome
NADH	nicotinamide adenine dinucleotide
QoL	quality of life
ROO	refined olive oil
SCFAs	short-chain fatty acids
